# Evaluating malaria case management at public health facilities in two provinces in Angola

**DOI:** 10.1186/s12936-017-1843-7

**Published:** 2017-05-03

**Authors:** Mateusz M. Plucinski, Manzambi Ferreira, Carolina Miguel Ferreira, Jordan Burns, Patrick Gaparayi, Lubaki João, Olinda da Costa, Parambir Gill, Claudete Samutondo, Joltim Quivinja, Eliane Mbounga, Gabriel Ponce de León, Eric S. Halsey, Pedro Rafael Dimbu, Filomeno Fortes

**Affiliations:** 1 Malaria Branch, Centers for Disease Control and Prevention, 1600 Clifton Road, Atlanta, GA 30329 USA; 2 President’s Malaria Initiative, Centers for Disease Control and Prevention, Atlanta, GA USA; 3 National Malaria Control Program, Ministry of Health, Luanda, Angola; 4 President’s Malaria Initiative, USAID, Washington, DC USA; 5 Management Sciences for Health, Luanda, Angola; 6 Management Sciences for Health, Washington, DC USA; 7 Field Epidemiology and Laboratory Training Program, Ministry of Health, Luanda, Angola; 8 President’s Malaria Initiative, USAID, Luanda, Angola

## Abstract

**Background:**

Malaria accounts for the largest portion of healthcare demand in Angola. A pillar of malaria control in Angola is the appropriate management of malaria illness, including testing of suspect cases with rapid diagnostic tests (RDTs) and treatment of confirmed cases with artemisinin-based combination therapy (ACT). Periodic systematic evaluations of malaria case management are recommended to measure health facility readiness and adherence to national case management guidelines.

**Methods:**

Cross-sectional health facility surveys were performed in low-transmission Huambo and high-transmission Uíge Provinces in early 2016. In each province, 45 health facilities were randomly selected from among all public health facilities stratified by level of care. Survey teams performed inventories of malaria commodities and conducted exit interviews and re-examinations, including RDT testing, of a random selection of all patients completing outpatient consultations. Key health facility readiness and case management indicators were calculated adjusting for the cluster sampling design and utilization.

**Results:**

Availability of RDTs or microscopy on the day of the survey was 71% (54–83) in Huambo and 85% (67–94) in Uíge. At least one unit dose pack of one formulation of an ACT (usually artemether–lumefantrine) was available in 83% (66–92) of health facilities in Huambo and 79% (61–90) of health facilities in Uíge. Testing rates of suspect malaria cases in Huambo were 30% (23–38) versus 69% (53–81) in Uíge. Overall, 28% (13–49) of patients with uncomplicated malaria, as determined during the re-examination, were appropriately treated with an ACT with the correct dose in Huambo, compared to 60% (42–75) in Uíge. Incorrect case management of suspect malaria cases was associated with lack of healthcare worker training in Huambo and ACT stock-outs in Uíge.

**Conclusions:**

The results reveal important differences between provinces. Despite similar availability of testing and ACT, testing and treatment rates were lower in Huambo compared to Uíge. A majority of true malaria cases seeking care in health facilities in Huambo were not appropriately treated with anti-malarials, highlighting the importance of continued training and supervision of healthcare workers in malaria case management, particularly in areas with decreased malaria transmission.

**Electronic supplementary material:**

The online version of this article (doi:10.1186/s12936-017-1843-7) contains supplementary material, which is available to authorized users.

## Background

As in many sub-Saharan African countries, febrile illness is the single largest cause of healthcare seeking in Angola, responsible for 35% of outpatient visits and 20% of all inpatient services in public health facilities [1]. Much of the febrile illness can be attributed to malaria, as a substantial proportion of Angolans are infected with malarial parasites at any given time, measured to be 13.5% in children under five in 2015–2016 [2, 3]. The entire country is endemic for malaria, although there is heterogeneity in malaria transmission, ranging from low, seasonal, epidemic-prone transmission in the dry south to high, year-round transmission in the wet, tropical north of the country. Despite having made substantial progress in rolling out malaria control interventions since the mid-2000s, there has been an increase in malaria cases in Angola in 2015 and 2016 as seen in routine data collected by the Angola National Malaria Control Programme (NMCP), with a concurrent increase in malaria mortality. Thus, ensuring appropriate diagnosis and treatment for malaria is a critical health priority in Angola.

The strategy to reduce the malaria burden in Angola has two primary components: the reduction of malaria incidence through prevention activities, including vector control, and prompt diagnosis and appropriate treatment of acute malaria cases [1]. Angola has adopted World Health Organization (WHO) recommendations for malaria case management. Artemisinin-based combination therapy (ACT) was introduced as first-line treatment for uncomplicated malaria in 2006. In 2009, the country adopted a policy of universal laboratory confirmation for all suspect malaria cases either with microscopy, restricted primarily to hospitals and health centres, or rapid diagnostic tests (RDTs), used at all levels. The three ACT currently in use for first-line treatment in Angola are artemether-lumefantrine, artesunate–amodiaquine and dihydroartemisinin–piperaquine. Pregnant women in the first trimester are treated with oral quinine. Severe malaria cases are treated, in order of preference, with intravenous artesunate, intramuscular artemether or intravenous quinine.

Provision of appropriate care for an acute malaria case is a multi-step process that can be divided into four consecutive steps [4]: the ill person must seek care (access), be treated appropriately by a healthcare provider complying with national treatment guidelines (compliance), adhere to the prescribed treatment (adherence), and respond appropriately to the treatment (efficacy). The coverage at each step can be estimated separately from multiple sources, and the overall effective coverage can be calculated as the product of the coverage at each step. Community-based surveys can provide estimates for the access and adherence steps, and data from anti-malarial resistance monitoring provide estimates of anti-malarial efficacy.

Perhaps the most difficult step to assess is the compliance step, where appropriate case management of malaria cases is provided at health facilities. Its implementation is a complex undertaking that requires the confluence of several key activities. Firstly, health facilities must have all the necessary commodities to diagnose and treat malaria, including a sufficient and continuous supply of quality-assured malaria tests, reagents for microscopy when applicable, and appropriate anti-malarials. Next, sufficient numbers of healthcare workers (HCWs) need to be trained in malaria case management, including use of RDTs, treatment, and when applicable, malaria microscopy. Next, HCWs need to be routinely supervised to ensure ongoing quality of service delivery and adherence to national treatment guidelines. Finally, accurate data on suspected and confirmed cases of malaria must be recorded and reported onward to ensure that decisions about malaria prevention and treatment can be targeted where they will have the greatest impact.

Assessment of the compliance step cannot be done from community-based surveys and necessitates collection of individual data at the health-facility level to characterize HCW performance for each of the steps in the case-management pathway. Evaluations in the form of systematic health facility surveys are a useful tool for monitoring the functioning of the different components of malaria case management implementation, especially in the context of changing malaria incidence and policies [5–7]. The Angola NMCP and the US President’s Malaria Initiative (PMI) conducted a malaria-specific health facility survey in the central province of Huambo in 2007, coinciding with the start of PMI support in the country. Serving as a baseline, the survey found overall poor practices by HCWs with substantial weaknesses in testing and treatment practices [8].

In early 2016, the Angola NMCP implemented a subsequent, malaria-focused health facility survey in Huambo Province, the site of the 2007 survey, and in Uíge Province, a PMI-supported province in the north of the country. The objective was to assess health facility readiness for malaria diagnosis and treatment and to evaluate the quality of malaria case management.

## Methods

**Fig. 1:**
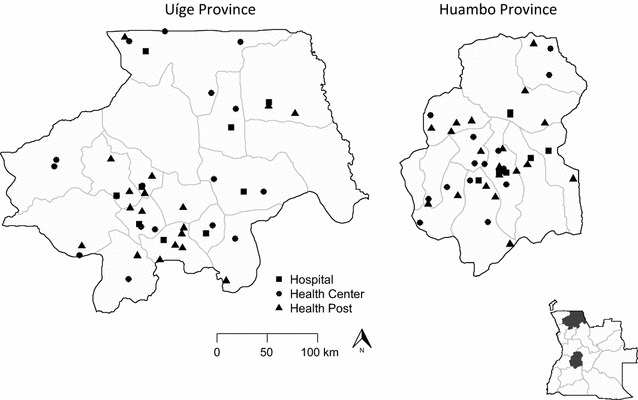
Location of health facilities visited during health facility surveys in Angola, 2016

A cross-sectional survey of health facilities was independently carried out in two provinces (Fig. 1): Huambo Province with meso-endemic-stable malaria transmission in the central highlands of Angola and Uíge Province with hyperendemic malaria transmission in northern Angola, an area covered with grassland savannahs interspersed with dense tropical forest. The survey took place in February 2016, at the peak of malaria transmission season in both provinces.

In each province, an exhaustive list of all public health facilities was obtained from the Provincial Directorate of Health, encompassing 230 health facilities in Huambo and 321 in Uíge. A total of 45 health facilities were randomly selected in each province, stratifying by health facility classification: nine hospitals, 18 health centres and 18 health posts. In each health facility, a maximum of five HCWs present during the visit were purposively chosen for interviews based on malaria case management workload. Additionally, a maximum of 20 patients were randomly invited to be interviewed and undergo a re-examination by a study clinician. All patients attending the outpatient department on the day of the visit were eligible for inclusion in the survey, independent of demographics or symptoms. The number of outpatient visits 1 week prior to the visit was abstracted from the health facility register, a sampling interval was determined, and randomly selected patients were invited to participate as they arrived. If the health facility saw fewer than 20 patients, all patients were invited to take part. In health facilities with separate paediatric and adult outpatient clinics, ten patients were chosen from each clinic.

The survey was powered to obtain a point estimate of the proportion of patients with an acute malaria episode treated with a first-line anti-malarial with a 95% confidence interval precision of 10%. Estimating that 40% of all patients would be suspect malaria cases, and assuming a 44% test positivity rate estimated from routine health facility data, a 75% treatment rate and a design effect of 2, the target sample size was calculated to be 820 patients per province.

## Data collection

In each province, four survey teams, each composed of a national-level supervisor and three interviewers, visited selected health facilities. The teams spent a full workday in each health facility, with data collection lasting 3 weeks per province. Survey teams carried out three primary activities: interviews with HCWs and facility directors; exit interviews and re-examinations of patients; and an inventory of 16 malaria commodities. Interviews were conducted using standardized questionnaires. During interviews with HCWs, interviewers enquired about HCW training, supervision and access to guidelines and job-aids.

Selected patients agreeing to participate in the survey were given a study identification card upon arrival at the health facility and were asked to present to the survey team once they completed their consultation and prior to leaving the health facility. During the exit interviews, interviewers asked about the history and symptoms of the current illness and asked patients to recall whether they were asked about fever by the HCW, whether they were tested for malaria during the consultation, what treatments they were prescribed, and what counselling they were provided by the HCW. Data on testing and treatment were verified through examination of any available patient cards and prescriptions and any drugs that were dispensed at the health facility. For the re-examination, survey teams took patient temperatures, performed an RDT (SD Bioline *P. falciparum*/*P. vivax*, Yongin, Republic of Korea), and provided artemether-lumefantrine, supplied by the Angolan NMCP, dosed according to weight, for RDT-positive patients that had not been prescribed appropriate anti-malarial treatment at the health facility.

At the end of the health facility visit, survey teams abstracted data from the health facility register for the selected patients and recorded the total number of outpatient visits during the day of the visit. All data collection was done on electronic tablets using SurveyCTO software (Dobility, Cambridge, USA).

## Variable definitions

Key health facility readiness indicators [9], including availability of key malaria commodities and HCW training and supervision, were calculated separately for each province. For each of the 16 malaria commodities, the proportion of health facilities managing each commodity (defined as regularly receiving and using the commodity) and the proportion stocked out on the day of visit (excluding expired commodities), were calculated. Any HCW that reported attending any malaria case management training was counted as having been trained in malaria case management.

Standard indicators for malaria case management [9], including the testing rate of suspect cases and treatment rate of confirmed cases, were calculated separately for each province. A suspect malaria case was defined as a patient complaining of fever or history of fever in the last 24 h, as recorded during the exit interview, or a with a temperature above 37.5 °C measured during the re-examination. A true case was defined as a suspect malaria case testing RDT-positive during the exit interview. Confirmed cases were defined as laboratory-confirmed cases based on testing by health facility staff that also tested RDT-positive during the exit interview. Correct management of a suspect malaria case was defined as testing by either RDT or microscopy at the health facility, and treatment with or prescription of a first-line anti-malarial with the correct dose only among patients testing positive during the re-examination (true positives). Patients with symptoms of severe malaria or general danger signs were excluded from the analysis.

The four key steps in the case-management pathway were evaluated separately to determine HCW performance: the proportion of patients who were asked about fever, spontaneously complained of fever, or had their temperature taken; the proportion tested by RDT or microscopy; the proportion treated or prescribed a first-line anti-malarial; and, the proportion receiving or prescribed the correct dose. To identify potential gaps in the case-management pathway for true malaria cases, the proportion of patients correctly managed at each step in the pathway was calculated, as was the cumulative proportion of patients correctly managed up to, and including, each step in the pathway.

For patients prescribed or given an ACT by the HCW, the quality of counselling provided by the HCW was assessed. Patients were asked to recall what instructions were given by the HCW regarding how to administer the ACT and under what circumstances they should return to the health facility. Patients were also asked to recite the dosing schedule for the prescribed ACT, including the number of tablets per dose, the number of doses per day, and the total number of days of therapy.

## Analysis

Health facility-level indicators were estimated adjusting by weighting by the inverse of the probability of health facility selection, and patient-level indicators were estimated adjusting by weighting by the inverse of the product of the probability of health facility selection and the probability of patient selection to adjust for utilization, using the R survey package [10].

Logistic regression was used to explore the relationship between a suspect malaria case being correctly managed and health facility type, availability of ACT and RDTs or microscopy, proportion of HCWs in the health facility trained in malaria case management, and the proportion of HCWs supervised in the last 6 months. Gender and age of the patient and the proportion of patients at the health facility testing positive for malaria during the re-examination (a measure of local malaria endemicity) were included as potential confounders.

Spatial heatmaps generated with Gaussian kernel smoothing were created for each province for six indicators: the proportion of patients with malaria, as determined during the re-examination; the proportion of suspect malaria cases correctly managed; the proportion of HCWs supervised in the last 6 months; the proportion of HCWs trained in malaria case management; the proportion of health facilities with ACT available; and the proportion of health facilities with RDTs or microscopy available.

All statistical analysis was done in R version 3.3.0 (R Foundation for Statistical Computing, Vienna, Austria).

## Ethical considerations

The survey was classified as a non-research, programme evaluation activity by Human Subjects Review Boards at the US Centers for Disease Control and Prevention and the Angolan Ministry of Health. All interviewed patients provided written informed consent.

## Results

Teams visited 89 out of 90 chosen health facilities, as one selected health facility in Huambo no longer existed, representing an error in the list provided. The final breakdown by health facility type (Table 1) did not match the sampling strategy since several health facilities in each province were misclassified on the lists provided by the provinces.

**Table 1:** Numbers and characteristics of health facilities, healthcare workers, and patients surveyed in Huambo and Uige Provinces, Angola, 2016

	n (%)		
	Huambo	Uíge	Total
Health facility	44	45	89
Hospital	8 (19)	9 (20)	17 (19)
Health centre	15 (35)	17 (38)	32 (36)
Health post	20 (47)	19 (42)	39 (44)
Healthcare workers interviewed	119	93	212
Patients interviewed	590	634	1224
<5 Years	222 (38)	162 (26)	384 (31)
5–15 Years	84 (14)	141 (22)	225 (18)
>15 Years	284 (48)	331 (52)	615 (50)
Female	367 (62)	396 (62)	763 (62)
Suspect malaria cases	360 (61)	430 (68)	790 (65)
True malaria cases^a^	51 (9)	242 (38)	293 (24)

^a^Defined as RDT-positive during survey re-examination

Availability of malaria tests and treatments on the day of the visit was similar in both provinces, with 71% (95% CI 54–83) of health facilities in Huambo and 85% (67–94) in Uíge with access to RDTs or microscopy, and 83% (66–92) of health facilities in Huambo and 79% (61–90) in Uíge with access to at least one formulation of an unexpired ACT in at least one unit dose package format (Table 2). However, stock-out rates of age-specific formulations were high in both provinces, ranging from 29 to 81% for artemether-lumefantrine, and 25 to 100%, skewed towards 100%, for artesunate-amodiaquine and dihydroartemisinin-piperaquine, which were only managed in a small subset of health facilities (see Additional file 1: Table S1). Availability of malaria commodities other than ACT and RDTs, such as severe malaria treatment, was also generally low in both provinces, with stock-out rates ranging from 25 to 100% (see Additional File 1: Table S1).

**Table 2:** Standard key indicators on health facility readiness for malaria care delivery, as assessed in health facility surveys in Huambo and Uíge Provinces, Angola, 2016

	Huambo		Uíge	
	%	95% CI	%	95% CI
Health facilities				
Offering any malaria diagnostic services	94	79–99	100	^a^
RDT	94	79–99	100	^a^
Malaria microscopy	21	12–33	4.5	2–9
Offering any malaria treatment	100	^a^	100	^a^
With RDT or malaria microscopy available on day of visit	71	54–83	85	67–94
With any formulation of ACT available on day of visit	83	66–92	79	61–90
With at least one HCW trained on RDT use	95	78–99	90	73–97
With at least one HCW trained on malaria microscopy	24	15–36	4.5	2–9
With at least one HCW trained on malaria treatment	99	96–100	98	93–99
With guidelines for diagnosis and treatment of malaria	96	90–98	51	34–68
HCWs trained in malaria case management	81	73–87	80	69–89
HCWs supervised in last 6 months	58	49–68	69	52–83

*RDT* rapid diagnostic test, *ACT* artemisinin-based combination therapy, *HCW* healthcare worker

^a^Confidence intervals undefined

A total of 119 HCWs were interviewed in Huambo and 93 in Uíge. Over 90% of health facilities in both provinces reported having at least one HCW trained in malaria case management and RDT use; 81% (73–87) of HCWs interviewed in Huambo and 80% (69–89) in Uíge reported receiving malaria case management training. In Huambo, 58% (49–68) of HCWs interviewed reported receiving a supervisory visit in the last 6 months, compared to 69% (52–83) in Uíge. Only 24% (15–36) of health facilities in Huambo and 4% (2–9) in Uíge reported having a trained microscopist (Table 2).

In Huambo, 590 patients were interviewed, with 360 (61%) meeting the criteria of a suspect malaria case (Table 1). In Uíge, 430 of 634 (68%) interviewed patients were suspect malaria cases. In Huambo, 14% (9–23) of suspect malaria cases were true malaria cases (tested positive by RDT during the re-examination), compared to 53% (45–60) in Uíge.

Among suspect malaria cases, only 30% (23–38) were tested by RDT or microscopy in Huambo, compared to 69% (53–81) in Uíge (Table 3). An additional 9% (6–14) of patients not meeting the suspect malaria case definition were tested in Huambo, increasing to 56% (39–72) in Uíge. In both provinces, testing with RDTs was substantially more common than microscopy. Patients undergoing testing at the health facility who were confirmed to have a positive RDT during the re-examination were likely to have been treated with a first-line anti-malarial, with treatment rates of 74% (52–88) in Huambo and 86% (74–93) in Uíge. A minority (2% in each province) of patients that had been tested at the health facility and were negative by RDT during the reexamination received anti-malarial treatment at the health facility. Overall, 27% (21–35) of suspect malaria cases were tested and were treated according to the test result in Huambo, compared to 59% (43–74) in Uíge. Among suspect malaria cases testing positive during the re-examination (true malaria cases), only 28% (13–49) had been given or prescribed a first-line anti-malarial with the correct dose in Huambo, compared to 60% (42–75) in Uíge.

**Table 3:** Standard key indicators on healthcare worker performance in malaria case management, as assessed in health facility surveys in Huambo and Uíge Provinces, Angola, 2016

	Huambo		Uíge	
	%	95% CI	%	95% CI
Suspect malaria cases receiving malaria test	30	23–38	69	53–81
<5 Years	30	23–38	82	65–92
RDT	25	18–33	81	64–91
Microscopy	5	2–13	1	0.2–4
≥5 Years	30	21–41	64	46–78
RDT	28	19–39	63	46–78
Microscopy	3	1–10	1	0.2–2
Confirmed malaria cases treated with appropriate anti-malarial	74	52–88	86	74–93
Suspect malaria cases negative for malaria^a^ but treated with anti-malarial	2	0.4–5.6	2	0.7–6.8
Suspect malaria cases not tested and treated with appropriate anti-malarial	1	0.3–4	7	2–23
Suspect malaria cases managed correctly^b^	27	21–35	59	43–74
True malaria cases appropriately treated^c^	28	13–49	60	42–75

*RDT* rapid diagnostic test

^a^During re-examination

^b^Tested and treated with first-line anti-malarial with correct dose only if positive

^c^Treated with first-line anti-malarial with correct dose

**Fig. 2:**
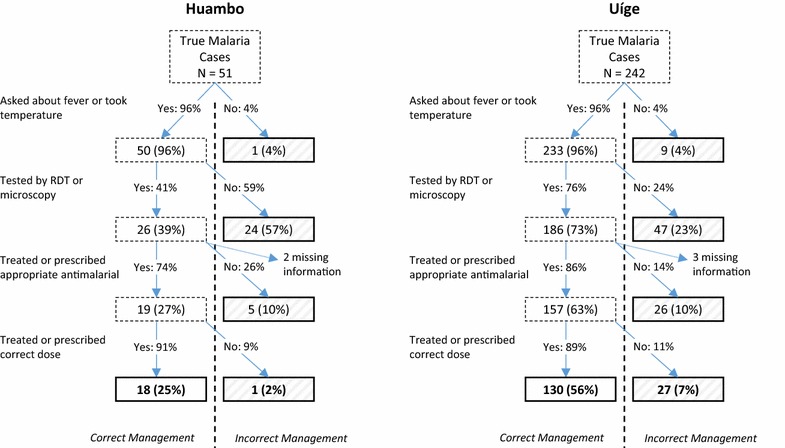
Healthcare worker performance for each step of the case-management pathway in acute malaria cases attending health facilities in Huambo and Uíge Provinces, Angola, 2016. Percentages in *boxes outlined in dashed lines* reflect cumulative proportion of patients managed correctly to that point. *Boxes outlined in bold* represent final categorization and percentages refer to final proportion of cases falling into each final categorization. All percentages are adjusted for cluster-sampling design

Analysis of the case-management pathway in these true malaria cases reveals that in Huambo the step least likely to happen was diagnostic testing. This step accounted for an overwhelming contribution to incorrect case management, with 57% of patients falling out of the correct case-management pathway at the testing step (Fig. 2). In contrast, in Uíge, performance along the entire pathway was more uniform, with each step being performed at a rate ranging from 76 to 96%.

In Huambo, the proportion of HCWs at the health facility reporting having received training in malaria case management was the only factor significantly associated with correct management of suspect malaria cases (adjusted OR: 2.7, 95% CI 1–8) (Table 4). Notably, correct malaria case management was not significantly associated with ACT or RDT availability. In contrast, in Uíge, availability of ACT was the strongest predictor of correct management of suspect malaria cases (adjusted OR: 7.2, 95% CI 3–20). HCWs were also more likely to manage suspect malaria cases incorrectly in older age categories in Uíge. In both provinces the majority of incorrect management of suspect cases of malaria (61% in Huambo and 73% in Uíge) took place in health facilities where both malaria testing and ACT were available.

**Table 4:** Factors associated with correct management of suspect malaria cases attending health facilities in Huambo and Uíge Provinces, Angola, 2016

Variable	Huambo		Uíge	
	Adjusted odds ratio	95% CI	Adjusted odds ratio	95% CI
Patient age				
<5 Years	Ref	–	Ref	–
5–15 Years	0.86	0.4–2	0.68	0.4–1
>15 Years	1.3	0.7–2	*0.4*	*0.2*–*0.7*
Patient sex				
Female	Ref	–	Ref	–
Male	0.89	0.5–1	0.66	0.4–1
Health facility type				
Hospital	Ref	–	Ref	–
Health centre	1.2	0.6–2	1.6	0.9–3
Health post	0.71	0.4–1	1.0	0.6–2
RDT or microscopy available on day of visit	1.7	0.7–5	2.5	1–7
ACT available on day of visit	0.55	0.3–1	*7.2*	*3*–*20*
Proportion of interviewed HCWs supervised in last 6 months	0.69	0.3–1	1.2	0.6–2
Proportion of interviewed HCWs trained in malaria case management	*2*.*7*	*1*–*8*	1.4	0.6–4
Proportion of patients testing true positive by RDT during re-examination	1.9	0.3–10	0.67	0.2–2

*RDT* rapid diagnostic test, *ACT* artemisinin-based combination therapy, *HCW* healthcare worker

A high proportion (above 85%) of patients prescribed an ACT in either province were instructed by the HCW on how to take the ACT and were able to correctly recite the dosing schedule (Table 5). However, the first dose of ACT was given at the health facility only 4% (95% CI 1–15) of the time in Huambo versus 45% (22–70) in Uíge. The HCW explained how to take artemether-lumefantrine, the most commonly prescribed ACT, with food only 34% (15–59) of the time in Huambo and 19% (11–31) of the time in Uíge. Less than half of all patients prescribed an ACT in both provinces reported being instructed to return to the health facility if the symptoms worsened or did not improve.

**Table 5:** Quality of counselling in patients prescribed an ACT as assessed during exit interviews in health facility surveys in Huambo and Uíge Provinces, Angola, 2016

	Huambo		Uíge	
	%	95% CI	%	95% CI
Given first dose at health facility	4	0.8–15	45	22–70
Given instructions on how to take ACT	85	57–96	89	76–96
Able to correctly recite dosing schedule^a^	90	78–96	85	77–91
Received instructions to				
Take with food				
AL	34	15–59	19	11–31
DP	29	12–56	98	88–100
Take on empty stomach				
AL	3	0.6–13	7	4–14
DP	29	12–56	0	–
Complete all doses	72	51–86	58	41–74
Return if worse	44	25–64	26	15–40
Return if no improvement	38	21–58	31	20–44

*ACT* artemisinin-based combination therapy, *AL* artemether-lumefantrine, *DP* dihydroartemisinin-piperaquine

^a^Correct number of tablets per dose, doses per day, and total duration of treatment. Calculated only for subset of patients prescribed the correct dose

There was substantial spatial heterogeneity in the proportion of patients presenting with malaria in both provinces, indicative of underlying spatial variation in malaria transmission (see Additional File 1: Figure S1, S2). There was also substantial heterogeneity in the performance on malaria indicators. In Uíge, the northeast of the province exhibiting very high malaria positivity also had the highest rates of ACT and RDT stock-outs, and the lowest rates of supervision and training.

## Discussion

The assessment revealed stark differences in the state of malaria case management in the two provinces. Over two-thirds of patients presenting with acute malaria infection in Huambo left the health facility without having been given or prescribed an appropriate anti-malarial. The poor performance of Huambo HCWs manifested in very low testing rates, with 30% of all febrile patients undergoing a laboratory diagnostic test for malaria, showing no improvement over the 31% testing rate measured in 2007 [8], despite the introduction and expansion of RDTs in the intervening time.

Despite an immense increase in RDT and ACT availability in sub-Saharan Africa in recent years, the overall proportion of malaria cases appropriately treated with anti-malarials is still low across the continent [11]. Although access to healthcare is an important factor, errors in malaria case management continue to be a major factor in the low population coverage. Commonly, the testing step in the case-management pathway is the main determinant of overall quality [12–15], and the results of this survey are no exception. The change to a policy of universal confirmation of suspected cases of malaria, made possible by the large-scale introduction of RDTs, has had a complex and variable effect on prescribing practices. In some settings, anti-malarial prescriptions have increased while in others it has decreased following the expansion of malaria testing [16]. In Huambo, for which two time points are available, 43% of true malaria cases that were not tested were still diagnosed with malaria (clinical diagnosis) in 2007. However, in 2016, empiric treatment with anti-malarials of suspect cases that were not tested was only 1%. Treatment of test-negative patients decreased from 60% in 2007 to 2% in 2016. However, because testing rates were measured to only be 30% in 2016, most true malaria cases were not treated with an ACT in Huambo, compared to a 49% rate of correct treatment of true malaria cases in 2007. This overall decrease in correct treatment of true malaria cases is a cautionary sign that the withdrawal of empiric treatment of malaria must be accompanied by a sustained effort to attain high testing rates [17].

In contrast to Huambo, malaria case management in Uíge was found to be relatively well functioning, with HCW performance at each step in the testing and treatment pathway above 75%. However, even with high performance at each individual step, overall effective coverage was low. Ultimately, only 56% of all malaria cases were correctly managed at all steps, an example of how overall low effective coverage can arise despite high coverage at individual steps in the context of interventions that require multiple sequential steps to be performed correctly [18]. Because there is currently no redundancy built into the system, for example in the form of empiric treatment, effective coverage will always be driven by the weakest steps in the case-management pathway.

The divergence of the two provinces should be interpreted in the context of historical malaria epidemiology in the two areas. Despite being in the highlands with moderate rainfall, Huambo routinely reported among the highest rates of malaria in any Angolan province in the last decade. As recently as 2010, Huambo reported over 500,000 annual cases of malaria [19]. However, by 2013, case numbers had fallen to just over 40,000 annual cases, 12% of the 2010 total, following intense vector control interventions and the scale-up of modern malaria control. This was reflected in the fact that only 51 malaria cases were identified out of 590 interviewed patients. This dramatic reduction in malaria cases might have caused a sense of complacency in HCWs in Huambo, removing suspicion of malaria and explaining the low observed testing rate. However, there are still pockets in Huambo with substantial numbers of malaria cases, particularly in the northeast corner of the province.

Appropriateness of case management of suspect malaria cases in Huambo was found to be associated with the proportion of HCWs trained in malaria case management at the health facility, which suggests that an effort to increase the total number of HCWs trained in malaria case management should improve this particular indicator. However, given the already high percentage of HCWs reporting being trained in malaria case management (81%), there is little room for this strategy to increase the rate of correct management. Improving ACT and RDT availability will also not completely address the root of the problem, as evidenced by the finding that most incorrect management of cases occurred in health facilities that had ACT and testing facilities available. Only an intervention that can change the mindset of HCWs to enforce the need to rule out malaria in all patients presenting with fever would be expected to substantially improve the overall outcome, since the decision to use available test kits was the weakest step in the process.

Uíge, in contrast to Huambo, has been reporting a relatively steady number of malaria cases to the NMCP over the last 5 years, approximately 150,000–250,000 cases per year, with no clear year-to-year trend. A different climate and economic situation have made malaria transmission in Uíge more recalcitrant than in Huambo, evidenced by the much larger proportion of malaria infections among interviewed patients. The survey suggests that HCWs in Uíge are largely cognizant of the role of malaria in the ill population they serve, with testing rates of suspect cases reaching 77%. Nevertheless, the weakest step in the case-management pathway was the diagnostic testing step. Since inappropriate case management was found to be associated with ACT stock-outs, improving the availability of malaria commodities should likely improve malaria care delivery. Nevertheless, this might prove challenging due to the inaccessibility of certain areas in the province. Certain health facilities were only reached after 16-h drives, with flooded, muddy and sandy roads hampering access. Notably, health facilities in the difficult-to-reach northeast of the province where the proportion of malaria cases was the highest were also the ones least likely to have ACT or RDTs on hand, or be staffed by HCWs trained in malaria case management or routinely supervised. Extra effort is needed to focus on areas with this profile to provide quality malaria care where it is most needed.

In both provinces, HCWs were generally effective in explaining the ACT dosing schedule to patients. However, only a minority of patients were instructed to take artemether-lumefantrine with food (34% in Huambo and 19% in Uíge). Artemether-lumefantrine should be administered with fatty foods to aid in absorption, and inappropriate administration of artemether-lumefantrine might lead to lower blood drug levels and increase the risk of treatment failure [20]. The importance of counselling patients to take artemether-lumefantrine with fatty foods should be emphasized in HCW training.

This survey was limited to some extent by a lower than expected sample size, having achieved only 75% of its target sample size. This was driven primarily by low patient flow in certain health facilities, particularly lower-level health facilities. As a result the overall average number of patients enrolled per health facility was 14 versus the expected 20. While the design effect for the indicator driving the sample size calculation, the proportion of patients with an acute malaria episode treated with a first-line anti-malarial, was 1.7 in Huambo, it was 10 in Uíge, substantially higher than the expected value of 2, and a major contributor to the large confidence intervals around the case management indicators in Uíge. Moreover, in Huambo, the low test positivity rate resulted in only 51 true malaria cases, reducing the precision of the survey’s estimates of certain case management indicators. Another limitation to the study was the choice of an exit interview strategy, which while accurate for indicators related to procedures such as RDT testing, can be less accurate for indicators related to counselling [21, 22].

This health facility survey was carried out during a difficult moment for malaria control in Angola. The fall in the price of oil and subsequent financial crisis coinciding with the loss of donor support from The Global Fund to Fight AIDS, Tuberculosis and Malaria (Global Fund) resulted in a weakening of malaria control efforts throughout Angola, starting in 2014 and continuing into 2016. Recent years have seen routine stock-outs of ACT and RDTs throughout Angola. This survey was performed shortly after an emergency shipment of malaria commodities from the Global Fund, and the ACT and RDT levels reported are likely not representative of their availability throughout the year. Yet, even during this survey, the stock-out rates of individual formulations of ACT were high, making treatment of different age categories even more difficult for HCWs.

In contrast to other sub-Saharan African countries reporting increases in malaria cases in recent years, Angola’s increase in morbidity has been accompanied by an increase in malaria mortality [19], likely aggravated by a yellow fever epidemic. Although this survey was not designed to examine clinical management of severe malaria cases, delayed or inappropriate management of uncomplicated malaria cases increases the likelihood of progression to severe disease and higher risk for malaria mortality. Moreover, the low availability of intravenous artesunate and intramuscular artemether means that most severe malaria cases are unlikely to be treated with the first-line therapies in Angola.

## Conclusion

Improvement of malaria case management is crucial to mitigate the effects of the current increase in malaria transmission in Angola. The results from this survey confirm modelling estimates that the vast majority of acute malaria episodes in Angola are not treated with appropriate anti-malarial therapy [11]. A main factor is low access to the formal healthcare sector, estimated to be 45% nationwide [1]; this could be mitigated by increasing the numbers of functional health centres or by establishing a programme for integrated community case management of childhood illness. However, the results reported here show that poor malaria case management at health facilities also contributes to the overall low coverage. The availability of malaria commodities and rates of appropriate treatment of the minority of malaria cases that do seek care in health facilities must be improved to decrease malaria morbidity and mortality in Angola.

## Additional file

**Additional file 1: Table S1.** Availability of malaria commodities during visits to health facilities in Huambo and Uíge Provinces, Angola, 2016. **Figure S1.** Geographic distribution of malaria case management and readiness indicators, Huambo Province, Angola, 2016; HF: health facility, RDT: rapid diagnostic test, ACT: artemisinin-based combination therapy. **Figure S2.** Geographic distribution of malaria case management and readiness indicators, Uíge Province, Angola, 2016; HF: health facility, RDT: rapid diagnostic test, ACT: artemisinin-based combination therapy.
