# Supplementary material for: Evaluating malaria case management at public health facilities in two provinces in Angola
Source: Malar J. 2017 May 3;16:186. doi: 10.1186/s12936-017-1843-7 (PMC5415823; doi:10.1186/s12936-017-1843-7)
Supplement: Supplementary file 1 — Additional file 1: Table S1. Availability of malaria commodities during visits to health facilities in Huambo and Uíge Provinces, Angola, 2016. Figure S1. Geographic distribution of malaria case management and readiness indicators, Huambo Province, Angola, 2016; HF: health facility, RDT: rapid diagnostic test, ACT: artemisinin-based combination therapy. Figure S2. Geographic distribution of malaria case management and readiness indicators, Uíge Province, Angola, 2016; HF: health facility, RDT: rapid diagnostic test, ACT: artemisinin-based combination therapy. [file 12936_2017_1843_MOESM1_ESM.docx]

**Supplemental Figure S1**. Geographic distribution of malaria case management and readiness indicators, Huambo Province, Angola, 2016; HF: health facility, RDT: rapid diagnostic test, ACT: artemisinin-based combination therapy

**Supplemental Figure S2**. Geographic distribution of malaria case management and readiness indicators, Uíge Province, Angola, 2016; HF: health facility, RDT: rapid diagnostic test, ACT: artemisinin-based combination therapy
